# Contrast gain control is a reparameterization of a population response curve

**DOI:** 10.1152/jn.00336.2024

**Published:** 2024-09-18

**Authors:** Elaine Tring, S. Amin Moosavi, Mario Dipoppa, Dario L. Ringach

**Affiliations:** ^1^Department of Neurobiology, David Geffen School of Medicine, University of California, Los Angeles, California, United States; ^2^Department of Psychology, David Geffen School of Medicine, University of California, Los Angeles, California, United States

**Keywords:** contrast adaptation, contrast gain, population coding, primary visual cortex

## Abstract

Neurons in primary visual cortex (area V1) adapt in varying degrees to the average contrast of the environment, suggesting that the representation of visual stimuli may interact with the state of cortical gain control in complex ways. To investigate this possibility, we measured and analyzed the responses of neural populations in mouse V1 to visual stimuli as a function of contrast in different environments, each characterized by a unique distribution of contrast values. Our findings reveal that, for a fixed stimulus, the population response can be described by a vector function **r**(*g_e_c*), where the gain *g_e_* is a decreasing function of the mean contrast of the environment. Thus, gain control can be viewed as a reparameterization of a population response curve, which is invariant across environments. Different stimuli are mapped to distinct curves, all originating from a common origin, corresponding to a zero-contrast response. Altogether, our findings provide a straightforward, geometric interpretation of contrast gain control at the population level and show that changes in gain are well matched among members of a population.

**NEW & NOTEWORTHY** The authors study the responses of neural populations in mouse primary visual cortex as a function of stimulus contrast. Measurements are performed in different “environments,” each characterized by a different distribution of contrast values. They find that responses across environments can be viewed as a reparameterization of a single contrast-response curve, offering a simple, geometric account of contrast gain control in neural populations.

## Graphical abstract

**Figure:**
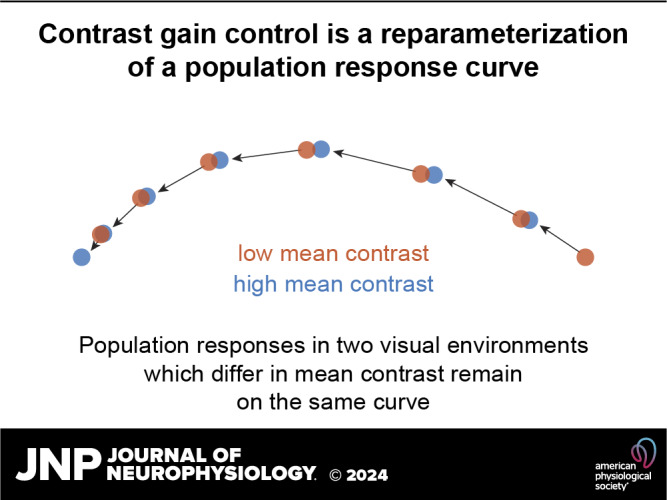

## INTRODUCTION

**Figure 1.**
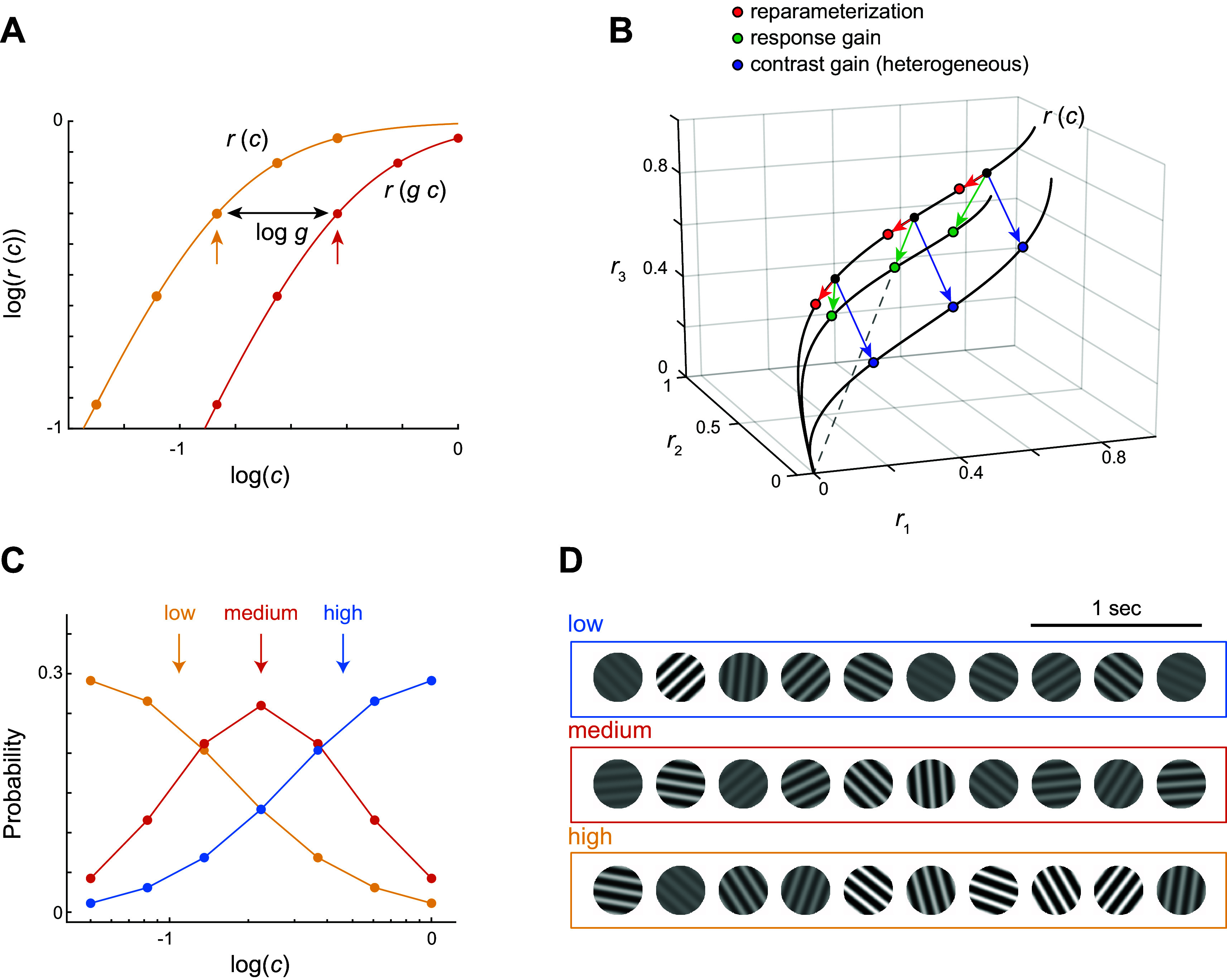
Gain control, hypothesis, and experimental design. *A*: early studies of contrast gain control measured the response of a single neuron in environments composed of 5 different contrast levels equally spaced along the log contrast axis (3). Yellow and orange dots represent the responses obtained in 2 different environments, and the geometric mean within each environment is indicated by the vertical arrows. Here and in the sequel, logarithms of contrast values are expressed in base 10, with 0 anchored at 100%. The result of such an experiment is that the contrast-response curves are shifted horizontally such that the geometric mean of the contrast aligns with the center of the curve. Thus, if *r*(*c*) expresses the responses in the yellow environment, then the responses in the orange environment with a higher mean contrast will be shifted to the right and can be described by *r*(*g c*). The horizontal shift between the curves is log *g*. Perfect adaptation occurs when *g* equals the ratio between the mean contrasts of the environments, but partial adaptation is also possible. *B*: different scenarios for how gain control could act at the population level. The responses of 3 hypothetical neurons in 1 environment at 3 different contrast levels are depicted by solid black dots. The dots lie on a curve *r*(*c*). Reparameterization predicts that switching to an environment with higher mean contrast would result in the responses shifting toward the origin (red arrows/dots). Response gain posits that responses will scale down toward the origin (green arrows/dots). The dashed line shows that the response will lie along the line joining the origin and the original population response. If neurons experience heterogeneous changes in contrast gain, the points ought to move to a different contrast-response curve (blue arrows/dots). *C*: definition of environments. Our experimental design consists of 3 environments defined by distinct contrast distributions on a fixed set of contrast levels covering the range from 5% to 100%. The geometric means of the distributions (vertical arrows) differ because of the different frequency of presentation of each contrast level. The contrast levels are the same for all environments [as opposed to the design from earlier studies (*A*)]. We used 3 distributions corresponding to high (blue), medium (orange), and low (yellow) mean contrast values. *D*: examples of stimulus sequences for the different experimental conditions. Within each environment, the contrast of gratings is drawn according to their respective distributions in *C*, while their orientation and spatial phase are uniformly distributed. The sequences were presented at a rate of 3 stimuli per second.

Neurons in primary visual cortex (area V1) respond to changes in the mean contrast of the environment by shifting their contrast-response function along the log contrast axis (1). The functional goal of such adaptation is to align the region of maximal sensitivity with the geometric mean of contrasts observed in the recent stimulus history, which can be viewed as an attempt to maximize information transmission (2). To illustrate this effect, consider *r*(*c*) to be the contrast-response function of a neuron measured in an environment with a specified average contrast (Fig. 1*A*, yellow curve; vertical arrow shows mean contrast). The contrast-response curve in a new environment with a higher mean contrast would be shifted to the right (Fig. 1*A*, orange curve). The transformed response can be described by *r*(*gc*), where *g* < 1 represents a reduction in contrast gain (1, 4, 5).

We hypothesize that such a relationship generalizes to neural populations. Specifically, we postulate that for a fixed stimulus the contrast-response function of a population is given by **r**(*g_e_c*), where **r** is a vector function, *c* is the stimulus contrast, and *g_e_* is a gain factor that depends on the distribution of contrasts in the environment. This gain factor is expected to decrease monotonically with the mean contrast of the environment. Mathematically, **r**(*g_e_c*) represents a linear reparameterization of a single contrast-response curve, which facilitates the understating of gain control from a geometric point of view. To appreciate the geometric interpretation, suppose that we measure the responses of the population at three contrast levels (Fig. 1*B*, black dots). These points will lie on the contrast-response curve of the population under the current environment, **r**(*c*) (Fig. 1*B*, black curve). Imagine we now proceed to switch the environment with one having a higher mean contrast. If contrast gain control operates under reparameterization, the responses of the population at the same contrast levels will merely shift along the curve toward the origin (Fig. 1*B*, red arrows and dots). From a coding perspective, the advantage of reparameterization is that a visual stimulus generates a single response curve that is invariant between environments. Assuming different stimuli generate different curves, the identification of stimulus by downstream areas is reduced to the question of whether a given population response lies on a given stimulus curve.

Implementing reparameterization requires the neurons in the population to adjust their gains by the same factor. If neurons respond to a change in the mean contrast of the environment by modifying their gains using significantly different factors, the responses will shift to an entirely different response curve, entangling the representation of visual stimuli with the state of cortical gain control (Fig. 1*B*, blue arrows and dots). In this scenario, the contrast response for a given stimulus depends on the state of gain control, complicating the decoding of visual information by downstream visual areas.

Finally, it may be possible for changes in the mean contrast of the environment to simply induce a change in the gain of the responses (6–9). In this model, the transformed response is given by *g_e_***r**(*c*), with *g_e_* representing, once again, a gain factor that decreases with the mean contrast of the environment. The signature of this “response gain” model is that the magnitude of a population vector changes between environments, while its direction remains constant (Fig. 1*B*, green arrows and dots). Such a coding strategy would be appropriate to generate an invariant representation of stimulus contrast, as one can then identify the direction of population vectors with the absolute contrast of a stimulus independent of the environment.

At first glance, the reparameterization hypothesis appears to be on shaky grounds, as V1 neurons exhibit considerable diversity when studied individually: some cells show robust changes in gain as a function of the mean contrast of the environment, while others do not adapt at all (1, 3). However, we must be careful in interpreting these studies, as responses were measured independently at each neuron’s optimal orientation and spatial and temporal frequencies. It is possible for the choice of stimulus parameters to have different effects on average cortical activity, which in turn may act as a normalization signal that controls neuronal gain (5, 10–12). Specifically, using stimulus parameters that are effective in driving a large fraction of the neurons in the population could generate a larger normalization signal than stimulus parameters that drive only a few. Thus, the diversity of stimulus parameters used in studying single neurons could be partly responsible for the large variability of gain changes observed in earlier datasets (1, 3). Instead, to address the reparameterization hypothesis directly and to circumvent problems in the interpretation of past data, we set out to measure and analyze the contrast response of a neural population to a fixed visual stimulus under different experimental conditions or “environments,” each associated with a unique distribution of contrast values with different means (Fig. 1, *C* and *D*).

To anticipate the results, we find that the population response to a fixed visual stimulus is well captured by a single vector function **r**(*g_e_c*). Moreover, we find that $g_{e}\;∼\;1/{\bar{c}}_{e}^{\;e}$ (with ϵ > 0), where ${\bar{c}}_{e}$ is the geometric mean of the contrast in the visual environment. Thus, gain control at the population level admits a simple description as a linear reparameterization of a contrast-response curve. A visual pattern can be identified with its unique contrast-response curve **r**(·), which is invariant across environments, thereby facilitating downstream decoding. Different visual patterns generate distinct curves, all originating from a common origin representing a zero-contrast response. These findings indicate that changes in contrast gain must be reasonably matched across all cells in a cortical population. Our results offer a simple, geometric interpretation of contrast gain control at the level of neural populations.

## MATERIALS AND METHODS

### Experimental Model and Subject Details

All procedures were approved by the University of California, Los Angeles (UCLA)’s Office of Animal Research Oversight (the Institutional Animal Care and Use Committee). In addition, the experiments complied with the guidelines set by the National Institutes of Health on animal research. A total of nine mice, four male and five female, aged postnatal day (P)35–56, were used. These animals were obtained as a cross between TRE-GCaMP6s line G6s2 (Jackson Laboratory, https://www.jax.org/strain/024742) and CaMKII-tTA (https://www.jax.org/strain/007004). There were no obvious differences in the results between male and female mice.

### Surgery

We measured cortical activity with two-photon imaging through cranial windows implanted over V1. Carprofen was administered preoperatively (5 mg/kg, 0.2 mL after 1:100 dilution). Mice were anesthetized with isoflurane (4–5% induction; 1.5–2% surgery). Core body temperature was maintained at 37.5°C via a feedback-controlled blanket. We coated the eyes with a thin layer of ophthalmic ointment during the surgery to protect the corneas. Anesthetized mice were mounted in a stereotaxic apparatus with blunt ear bars placed in the external auditory meatus. A section of the scalp overlying the two hemispheres of the cortex was then removed to expose the skull. The skull was dried and covered by a thin layer of Vetbond, and an aluminum bracket was affixed with dental acrylic. The margins were sealed with Vetbond and dental acrylic to prevent infections. A high-speed dental drill was used to perform a craniotomy over monocular V1 on the left hemisphere. Special care was exercised to ensure that the dura was not damaged during the procedure. Once the skull was removed, a sterile 3-mm-diameter cover glass was placed on the exposed dura and sealed to the surrounding skull with Vetbond. The remainder of the exposed skull and the margins of the cover glass were sealed with dental acrylic. Mice were allowed to recover on a heating pad, and once awake they were transferred back to their home cage. Carprofen was administered postoperatively for 72 h. We allowed mice to recover for at least 6 days before conducting the first imaging session.

### Two-Photon Imaging

Imaging sessions took place 6–9 days after surgery. Procedures were identical to those described previously (13). Mice were positioned on a running wheel and head-restrained under a resonant, two-photon microscope (Neurolabware, Los Angeles, CA). The microscope was controlled by Scanbox acquisition software and electronics (Scanbox, Los Angeles, CA). The light source was a 920-nm-excitation beam generated by a Coherent Chameleon Ultra II laser (Coherent Inc., Santa Clara, CA). We used a ×16 water immersion objective for all experiments [Nikon; 0.8 numerical aperture (NA), 3-mm working distance]. The microscope frame rate was 15.6 Hz (512 lines with a resonant mirror at 8 kHz). The field of view was 730 µm × 445 µm in all sessions. The objective was tilted by 8–10° to be approximately normal on the cortical surface. Images were processed with a pipeline consisting of image registration, cell segmentation, and signal extraction using suite2p (14). A custom deconvolution algorithm consisting of linear filtering followed by half-rectification and a power function was used (15).

### Visual Stimulation

A Samsung CHG90 monitor, positioned 30 cm in front of the animal, was used for visual stimulation. The screen was calibrated with a Spectrascan PR-655 spectro-radiometer (Jadak, Syracuse, NY), generating gamma corrections for the red, green, and blue components via a GeForce RTX 2080 Ti graphics card. Visual stimuli were generated by a custom-written Processing 4 sketch using OpenGL shaders (see http://processing.org). At the beginning of each experiment, we obtained a coarse retinotopy map of the cortical section under study (16). The center of the aggregate population receptive field was used to center the location of our stimuli in these experiments. Stimuli were presented within a circular window with a radius of 25°.

We used a sequence of flashed, sinusoidal gratings presented at a rate of 3/s for stimulation. The spatial frequency was fixed at 0.04 cycles/°, matching the average preference of the V1 population (17). Sequences were presented in blocks representing one among three possible environments with different contrast distributions (Fig. 1*C*). The distributions were truncated log-normal sampled on a discrete set of seven contrast levels 5% × ξ*^q^* for *q* = 0, 1,…, 6 and ξ = 1.6475. Thus, contrast levels were equally spaced in logarithmic steps. The geometric means of the contrast in the three environments were ${\bar{c}}_{L}$ = 10.9%, ${\bar{c}}_{M}$ = 22.3%, and ${\bar{c}}_{H}$ = 46.0% (Fig. 1*C*, vertical arrows), which we refer to as the low (*L*)-, medium (*M*)-, and high (*H*)-contrast environments. Thus, the average contrasts of the environments are spaced by an octave. Note that although the mean contrasts in the environments differ, their range is the same, as opposed to the experimental design used in earlier studies (Fig. 1*A*). This allows us to compute the responses over the entire range of contrasts in all environments. Stimulus sequences were generated by uniformly drawing the orientation and spatial phase of the grating, while drawing the contrast from the corresponding environment distribution (Fig. 1*D*). All six permutations of {*L*, *M*, *H*} environments were presented in a randomized order, leading to a total of 18 experimental blocks. Each block was presented for 5 min, for a total of 900 stimuli per block. Each environment appeared six times during the session, resulting in 5,400 stimuli per environment. A 1-min blank screen was presented between blocks. The presentation of each grating was signaled by a transistor-transistor logic (TTL) pulse sampled by the microscope. As a precaution, we also signaled the onset of the stimulus by flickering a small square at the corner of the screen, and the signal of a photodiode placed at that location was sampled by the microscope as well.

### Definition of Population Responses

For each environment e∈{L,M,H}, and orientation θ, we calculated the mean response, **r***_e_*(θ,*c*,*T*), averaged over spatial phase, *T* microscope frames after the onset of the stimulus. Here, e∈{L,M,H} is one of the environments (Fig. 1*B*), and θ represents the orientation of the grating and *c* its contrast. The response averaged across all orientations is denoted by **r***_e_*(*c*,*T*). As we will see, the largest response magnitude is obtained for *e* = *L* and *c* = 100%. Thus, we define the optimal time to peak, *T*_opt_, as the one for which the Euclidean norm of *r_L_*(100%,*T*) attained its peak after stimulus onset. Across all our sessions we found *T*_opt_ = 287 ± 33 ms (mean ± 1 SD, *n* = 17). We define **r***_e_*(θ,*c*) = *r_e_*(θ,*c*,*T*_opt_) and, similarly, **r***_e_*(*c*) = *r_e_*(*c*,*T*_opt_). The Euclidean norm of these vectors is denoted by *r_e_*(θ,*c*) and *r_e_*(*c*), respectively. The temporal response of the population norm to a stimulus returns to baseline at ∼500 ms after stimulus onset (data not shown). Therefore, no major (linear) contribution from stimuli earlier in the sequence is expected at *T*_opt_, as the response to the prior stimulus, present 333 ms earlier, has already returned to baseline.

There is a technical point concerning the estimate of population norms that deserves attention. We want to estimate the Euclidean norm *r* of the mean population response to *m* trials of a stimulus in a population of *d* independent neurons. The response of the neurons in any one trial is the realization of a random variable *r_i_*, with *i* = 1, …, *d*. The actual response of neuron *i* to the stimulus in trial *k* is denoted by $r_{i}^{k}$. If we let $\mu_{1}^{i}\;$ represent the mean response of the *i*th neuron, we want to find an estimate of $\left\|\mu \right\|=\sqrt{\sum_{i}{\left(\mu_{1}^{i}\right)}^{2}}$ from the data $\left\{r_{i}^{k}\right\}$.

A reasonable way to proceed is to estimate the squared norm of the population response as ${\left\|\mu \right\|}^{2}\approx {\sum_{i=1}^{d}\left(\frac{1}{m}\sum_{k=1}^{m}r_{i}^{k}\right)}^{2}$. However, it is easy to see that this estimate is biased. First, let us consider the inner term, which expands to

$${\left(\frac{1}{m}\sum_{k=1}^{m}r_{i}^{k}\right)}^{2}=\frac{1}{m^{2}}\left({\sum_{k=1}^{m}\left(r_{i}^{k}\right)}^{2}+\sum_{k\neq l=1}^{m}r_{i}^{k}r_{i}^{l}\right).$$

What would be the average value of this quantity if we were to repeat the experiment many times? If we take expected values on both sides of the equation we obtain

$$E\left\{{\left(\frac{1}{m}\sum_{k=1}^{m}r_{i}^{k}\right)}^{2}\right\}=\frac{1}{m^{2}}\left(m\mu_{2}^{i}+m\left(m-1\right){\left(\mu_{1}^{i}\right)}^{2}\right)=\frac{\mu_{2}^{i}-{\left(\mu_{1}^{i}\right)}^{2}}{m}+{\left(\mu_{1}^{i}\right)}^{2}=\frac{\text{var}\{r_{i}\}}{m}+{\bar{r}}_{i}^{2}.$$

Here, $\mu_{1}^{i}=E\left\{r_{i}\right\}={\bar{r}}_{i}$ represents the mean of *r_i_*, and $\mu_{2}^{i}=E\left\{r_{i}^{2}\right\}$ is its second moment. Thus, on average, there is a bias term $\frac{\text{var}\{r_{i}\}}{m}$, which depends on the number of trials. To correct for it, we calculate the sample variance and subtract the term $\frac{\text{var}\{r_{i}\}}{m}$ from ${\left(\frac{1}{m}\sum_{k=1}^{m}r_{i}^{k}\right)}^{2}$. Finally, to obtain an estimate of the norm, we add all the terms across neurons and take the square root. Such bias correction was applied to the calculation of *r_e_*(θ,*c*).

### Bézier Curve Fit to Contrast Response Data

For a fixed visual pattern, we obtain the mean response to 7 different contrast levels in 3 different environments (a total of 21 points). As we show in results, these points can be projected into the first two principal components without major geometric distortions. These data appear to lie on a single curve. To characterize the shape of such a curve, we fit a quadratic Bézier curve as follows. First, the data are normalized so that the response with minimum norm is mapped to (0,0), while the response with the largest norm is mapped to (0,1). We achieve this with a similarity transformation, which does not distort the shape of the curve. A quadratic Bézier curve has 3 control points. We choose the first one to align with the origin, **p**_0_ = (0,0) and the third one to be **p**_2_ = (0,1). The only free parameter left is the point **p**_1_, which leads to the Bézier curve: **B**(*t*) – 2*t*(1 – *t*)**p**_1_ + *t*^2^(0,1). For a given choice of **p**_1_ we can compute the minimum distance from each data point to the curve and define the error of the fit as the mean square distance across all points. Then, we minimize the error as a function of **p**_1_, using MATLAB’s fminsearch. There is nothing particularly special about the use of Bézier curves; other interpolation methods could have worked as well to illustrate that the responses lie along a smooth curve (see Fig. 3).

### Rigor and Reproducibility

We conducted experiments by independently measuring the adaptation of V1 populations in *n* = 17 independent sessions. Linear models were fitted to the data with MATLAB’s fitlm function. The goodness of fit of linear models was evaluated using the coefficient of determination, *R*^2^. As the study did not involve different groups undergoing different treatments, there was no need for randomization or blind assessment of outcomes. We processed all putative neurons selected by suite2p (14) and selected only those that responded significantly to visual stimulation. This was done by calculating the ratio between the response of neurons at the optimal time for the population, *T*_opt_, and their baseline response just before the onset of stimulation. We then selected neurons for which such ratio was > 8. No selection took place with respect to the tuning of neurons for orientation: both cells with good and poor orientation selectivity were included. The median number of cells in our populations was 110, with the first and third quartiles at 210 and 335, respectively.

## RESULTS

We begin by providing low-dimensional visualizations of the geometry of **r***_e_*(θ,*c*) using principal component analysis (PCA). These analyses reveal some potential features of gain control, including the possibility that gain control acts as a reparameterization of a single contrast-response curve. As low-dimensional visualizations can incur geometric distortions, subsequent analyses are performed in native response space. In this context, we investigate the structure of the pairwise Euclidean distance matrix $d(r_{e_{1}}\left(c_{1}\right),r_{e_{2}}(c_{2}))$ between responses across environments. To test the reparameterization hypothesis, we show that given two different environments *e*_1_ and *e*_2_, where the geometric mean of contrast in *e*_2_ is lower than in *e*_1_, we can find a constant *g* < 1 such that $d(r_{e_{1}}\left(c\right),r_{e_{2}}(g\;c))\approx 0$. This finding implies that $r_{e_{1}}(\cdot )$ and $r_{e_{2}}(\cdot )$ lie approximately on a single response curve, the hallmark of reparameterization. We then provide a statistical model for the dependence of the response magnitude *r_e_*(*c*) with changes in the environment, showing that $g_{e}\;∼\;1/{\bar{c}}_{e}^{\;e}$, where ${\bar{c}}_{e}$ is the geometric mean of the contrast in the visual environment. We also consider and reject the response gain model as a good contender to explain the data. Finally, we relate the properties of the population to those of single neurons and provide an estimate of the dispersion of gain control in a population of V1 neurons.

### Low-Dimensional Visualization of Population Responses

**Figure 2.**
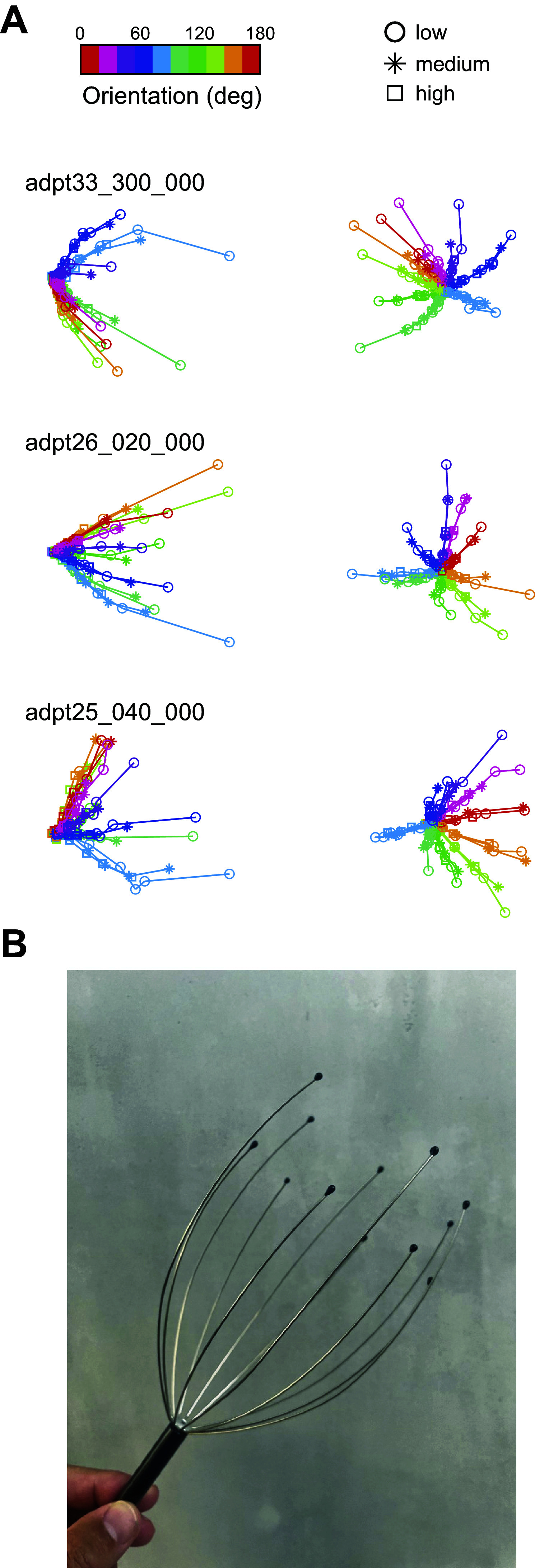
Low-dimensional visualization of population responses. *A*: each row corresponds to data from a different experimental session, showing 2 views of the projection of the data into the first 3 principal components. Each color corresponds to gratings of different orientations (color bar, *top*), and each symbol represents data for environments with different mean contrasts (circles = low, asterisks = medium, squares = high). The contrast responses for a fixed orientation across different orientations all lie approximately on the same curve. Mean responses for low contrasts converge into a point. *B*: a visual that captures the overall structure of the data. Each “arm” of the scalp massager represents a contrast-response curve for a fixed orientation. Changing environments causes a shift in the mean responses along the arm, which implies that gain control is a reparameterization of a single curve.

As a starting point, we used principal component analysis to examine the structure of **r***_e_*(θ,*c*) in three dimensions (Fig. 2*A*). Different colors are used in Fig. 2*A* to show data from different orientations; each environment is assigned a different symbol (low = circles, medium = asterisks, high = squares). For a fixed environment and orientation, straight lines join data points at adjacent contrast levels. The resulting curves show the shape of **r***_e_*(θ,*c*) as a function of *c* for the different environments and stimulus orientations.

Some salient features in the structure of the data catch the eye. First, for any given orientation, the contrast-response functions across different environments overlap substantially: all symbols representing the data for a single orientation at different contrast levels and environments appear to lie approximately on a single curve. This is consistent with the idea that gain control acts to reparametrize the population response (Fig. 1*B*). In other words, changing the environment only shifts the data along the curve and does not move them “off the manifold” associated with a given orientation (18). Second, different orientations generate different curves emanating from a common origin. This type of structure is expected from the orientation tuning of cortical neurons and the fact that, as contrast is decreased, we anticipate the responses at all orientations to converge to the response corresponding to a zero-contrast “origin.” Third, the contrast-response curves resemble straight rays at low contrast values but show clear curvature at moderate to high contrast values. The curvature is mostly visible in data from the low-contrast environment, which also generates the responses with the largest magnitudes. One can think of the geometry of the responses as resembling a scalp massager, with each wire representing the “arms” generated by stimuli at different orientations (Fig. 2*B*). The arms remain invariant with changes of the environment. Of course, these observations should be interpreted with care, as the fraction of variance captured by a projection into the first three principal components is only about half of the total (0.54 ± 0.056, mean ± 1 SD).

### Contrast Responses for a Fixed Stimulus across Different Environments Lie on a Curve

**Figure 3.**
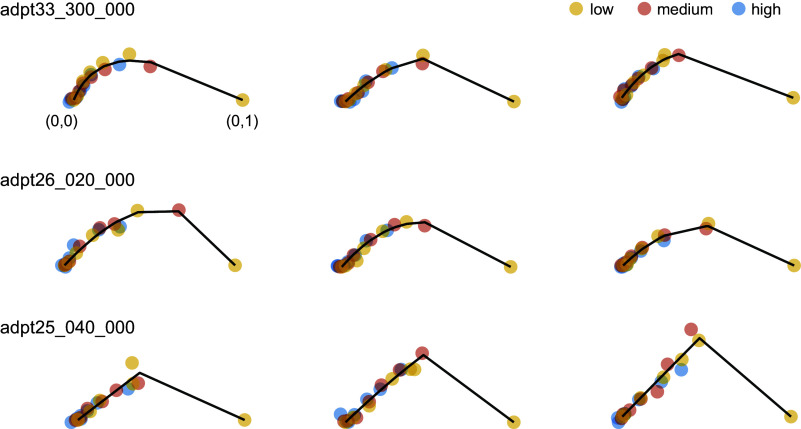
Contrast responses to a fixed visual pattern across different environments lie approximately on a 2-dimensional (2-D) curve. Each row shows the structure of some “arms” in 1 experimental session. For each arm, the data points represent the projection of the mean responses at a fixed orientation for varying levels of contrast for the 3 environments (low, medium, and high contrast) onto the first 2 principal components. Each environment is coded by a different color. Only 3 orientations per session are shown. Each arm is normalized so that the response with minimum norm is mapped to (0,0) and the one with largest response to (0,1). Dark segments join the locations on the Bézier curve fit that are closest to the data.

Our visualization of the data by principal component analysis (PCA) in three dimensions (3-D) is likely to be dominated by the need to capture the disparate responses evoked by different orientations. Instead, to focus on the analysis of the shape of the contrast-response functions, we performed PCA on the individual arms of the dataset, each representing the responses in all 3 environments for 7 contrast levels at a single orientation. Our analyses show that projecting the data into the first two principal components accounts for 0.83 ± 0.033 (mean ± 1 SD) of the total variance of the arms, reasonably capturing their shape (Fig. 3). Thus, contrast responses for a fixed orientation lie mostly on a plane. The projected data points in two dimensions (2-D) were normalized using a similarity transformation, such that the response with the smallest norm was mapped to (0,0) and the one with largest norm mapped to (0,1). This was done to allow the comparison of the shape of contrast-response functions for different orientations.

A key observation is that data points for each orientation tend to fall along a single curve (Fig. 3). The largest responses (farther from the origin) are obtained for the highest contrast level in the low-contrast environment. Switching from the low-contrast environment to a medium-contrast environment shifts the points along the curve toward the origin at (0,0). The same occurs as we move from the medium-contrast environment to the high-contrast environment. This finding is consistent with the hypothesis that gain control serves to reparametrize the population responses. Moreover, as we will see below, this reparameterization has a simple dependence on the mean contrast of the environment. We note that in some experiments the data lay almost entirely along a ray except for the point at the highest contrast in the low-contrast environment (Fig. 3, *bottom*). This likely resulted from our experimental design undersampling the 60–100% contrast range, a limitation we plan to correct in future studies.

### Testing Reparameterization Using Pairwise Distances in Native Space

Next, to eliminate any possible distortions in the structure of the data incurred by dimensionality reduction methods, we tested the reparameterization hypothesis directly in native space. By “native space” we mean the Euclidean, *d*-dimensional space, where the response vectors live. We achieve this by studying the structure of pairwise distance matrices $d(r_{e_{1}}\left(c_{1}\right),r_{e_{2}}(c_{2}))$, as follows. Let us assume, without loss of generality, that the mean of contrast in *e*_2_ is lower than in *e*_1_. Thus, responses in *e*_2_ at any one contrast are larger than the ones obtained in *e*_1_. The linear reparameterization hypothesis predicts that the response to *c*_1_ in *e*_1_ can be matched by reducing the contrast in *e*_2_ by a fixed gain factor, $d(r_{e_{1}}\left(c\right),r_{e_{2}}(g\;c))\approx 0$. Indeed, when plotting $d(r_{\text{med}}\left(c_{1}\right),r_{\text{low}}(c_{2}))$ and $d(r_{\text{high}}\left(c_{1}\right),r_{\text{med}}(c_{2}))$ we observe that minimum distances fall approximately on a diagonal parallel and displaced from the identity line. As contrast axes are logarithmic, such a shift represents a multiplicative effect of the environment on contrast, the signature of gain control. The same effect is seen in the structure of *d*(**r**_high_(*c*_1_),**r**_low_(*c*_2_)). Here, the shift of the diagonal doubles, matching a doubling in the ratio between the mean contrasts of the environments. Altogether, we conclude that for any two environments we can find a factor *g* such that $d(r_{e_{1}}\left(c\right),r_{e_{2}}(g\;c))\approx 0$. One caveat is that both measurement noise and the coarse sampling of contrast values provide a lower bound on how close to zero this value can get. In these analyses, distances were normalized by the diameter of the dataset (the maximum distance between any 2 responses), and the minimum distances obtained were 0.096 ± 0.022 (mean ± 1 SD).

### Dependence of Gain on the Mean Contrast of the Environment

**Figure 4.**
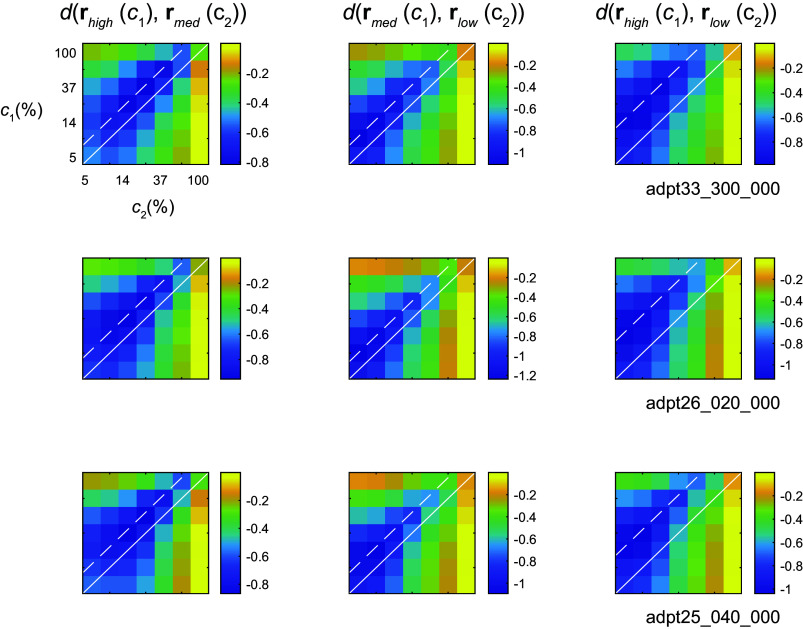
The structure of distance matrices is consistent with reparameterization. Each row, representing data from different sessions, shows the Euclidean distance between population responses across different environments. *Left*: the distance between responses in high- and medium-contrast environments. *Center*: between medium- and low-contrast environments. *Right*: between high- and low-contrast environments. Distance matrices were computed for each orientation separately and then averaged across orientations. Distances are normalized to the diameter of the dataset and displayed on a logarithm (base 10) scale. We observe that distance minima lie on a diagonal parallel to the identity line. The dashed white line represents the location of the diagonal on which the average distances reach a minimum. The solid white line is the identity line. Such diagonal structure is consistent with the linear reparameterization hypothesis.

**Figure 5.**
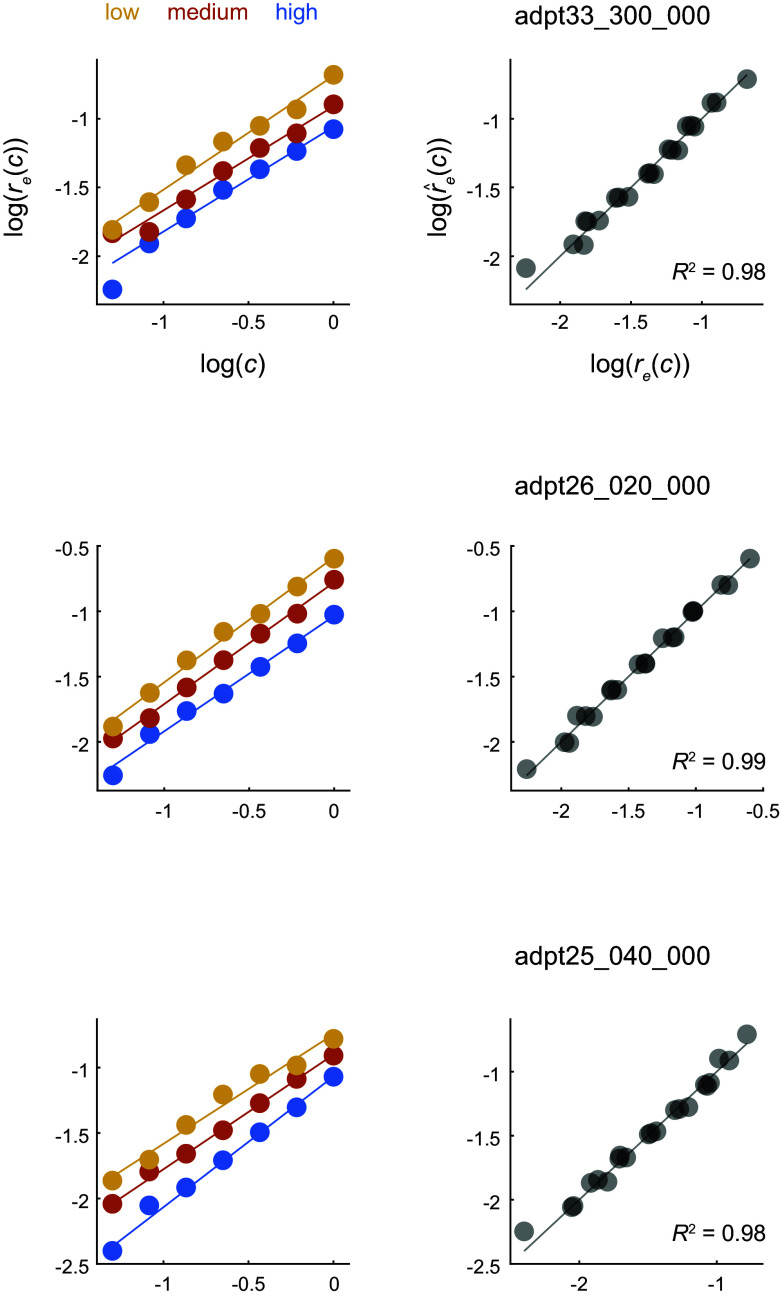
Dependence of gain on the mean contrast of the environment. Each row represents data from sessions matching those in prior figures. *Left*: the magnitude of the responses as a function of contrast for the different environments. The plot is in double logarithmic axes. The solid lines represent the best linear fits to the data from each environment (fit independently for each environment). The lines have approximately the same slope and are shifted in about equal amounts. This suggests that the entire dataset may be captured by the model ${\hat{r}}_{e}\left(c\right)=A\;{\left(\frac{c}{{\bar{c}}_{e}\epsilon }\right)}^{\delta }$. *Right*: the fit of such a model to the data. The quality of the fits is very good, with *R*^2^ values ∼0.98. The solid gray line represents the identity line.

How does the gain *g* change as a function the mean contrast of the environment? Although the location of the diagonals along which the distance is minimum offers one approach (Fig. 4), it is limited by the coarse sampling of contrast in the data. Instead, we considered an alternative approach that relies on the comparison of response magnitudes between environments. Note that if $d(r_{e_{1}}\left(c\right),r_{e_{2}}(g\;c))=0$, then the magnitudes must satisfy $r_{e_{1}}\left(c\right)=r_{e_{2}}(g\;c)$. Therefore, in analogy to the study of single neurons, we should see a horizontal shift in the magnitude of population responses between environments by log *g* (Fig. 1*A*). Indeed, when the population magnitudes are plotted as a function of contrast in double logarithmic axis, they appear as shifted lines (Fig. 5). This analysis also replicates our previous finding that the magnitudes of population responses are a power law of contrast, *r*(*c*) ∼ *c*^δ^ (13). Unfortunately, the shift in the population norm is ambiguous, as it could potentially be interpreted as either a horizontal or vertical shift of a line, corresponding to changes in contrast gain, response gain, or a mixture of both (8, 19). However, as we argue below, a horizontal shift is the only option consistent with the structure of the geometry of the responses. For the moment, let us take this point for granted and describe the transformation observed as changes in contrast gain. Note that magnitudes of the shifts are approximately equal as we move from low-contrast to medium-contrast and from medium-contrast to high-contrast environments, representing equal steps in log contrast (Fig. 1*C*). Thus, it is natural to put forward a gain control model ${\hat{r}}_{e}\left(c\right)=A\;{\left(\frac{c}{{\bar{c}}_{e}^{\epsilon }}\right)}^{\delta }=A\;{\bar{c}}_{e}^{-\delta \epsilon }c^{\delta }$, where ${\bar{c}}_{e}^{\epsilon }$ is the geometric mean of the contrast in *e*. As it turns out, this model performs extremely well (Fig. 4; *R*^2^ = 0.976 ± 0.01, mean ± 1 SD, *n* = 17). Across all sessions, we obtain δ = 0.81 ± 0.092 and ϵ = 0.68 ± 0.06 (mean ± 1 SD). Altogether, we conclude that the response of the population to a fixed visual pattern in environment *e* is given by **r***_e_*(*g_e_c*) with $g_{e}=\frac{1}{{\bar{c}}_{e}^{\epsilon }}$.

### The Response Gain Model Is Inconsistent with the Geometry of the Data

**Figure 6.**
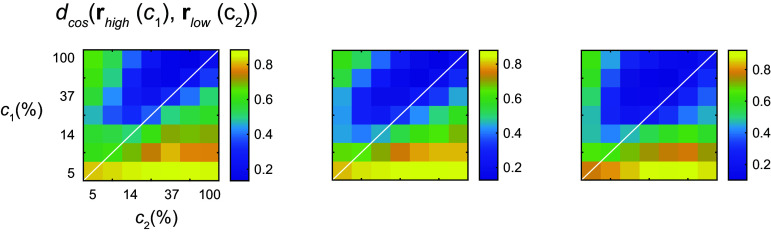
The response gain model, where the directions of the responses are invariant across environments, is inconsistent with the geometry of the data. Each panel shows the cosine distance between responses in the high- and low-contrast environments in independent sessions. Whereas response gain predicts that distances should be near 0 along the main diagonal, we observe the minima off the main diagonal in parallel with the behavior of the Euclidean distance matrices (Fig. 4).

Let us now go back to discuss the ambiguity between changes in response versus contrast gain in the population magnitude data (Fig. 5). The response gain model is represented by the relationship *g_e_***r**(*c*), where the output gain is modulated by the environment (Fig. 1*B*). This implies that, for any given contrast, the direction of population response should not change across environments: only its magnitude does. Thus, one would predict that the cosine distance matrix $d_{\text{cos}}(r_{e_{1}}\left(c_{1}\right),r_{e_{2}}(c_{2}))$ should be zero along its main diagonal, when *c*_1_ = *c*_2_. Instead, we observe that the directions of the population vectors across two environments are most similar at different contrast values (Fig. 6), in a way that parallels the behavior of the Euclidean distance matrix (Fig. 4). Thus, even though the magnitude data are ambiguous, we safely conclude that the geometry data reject the response gain model.

### Assessment of the Dispersion of Gain Control in a Population

In principle, reparameterization strictly holds only when the changes in gain in the population are identical. Of course, some degree of variability is expected in the data. Here, we assess the degree of gain control dispersion across neurons for a fixed visual stimulus across environments and how it relates to the analyses at the population level.

**Figure 7.**
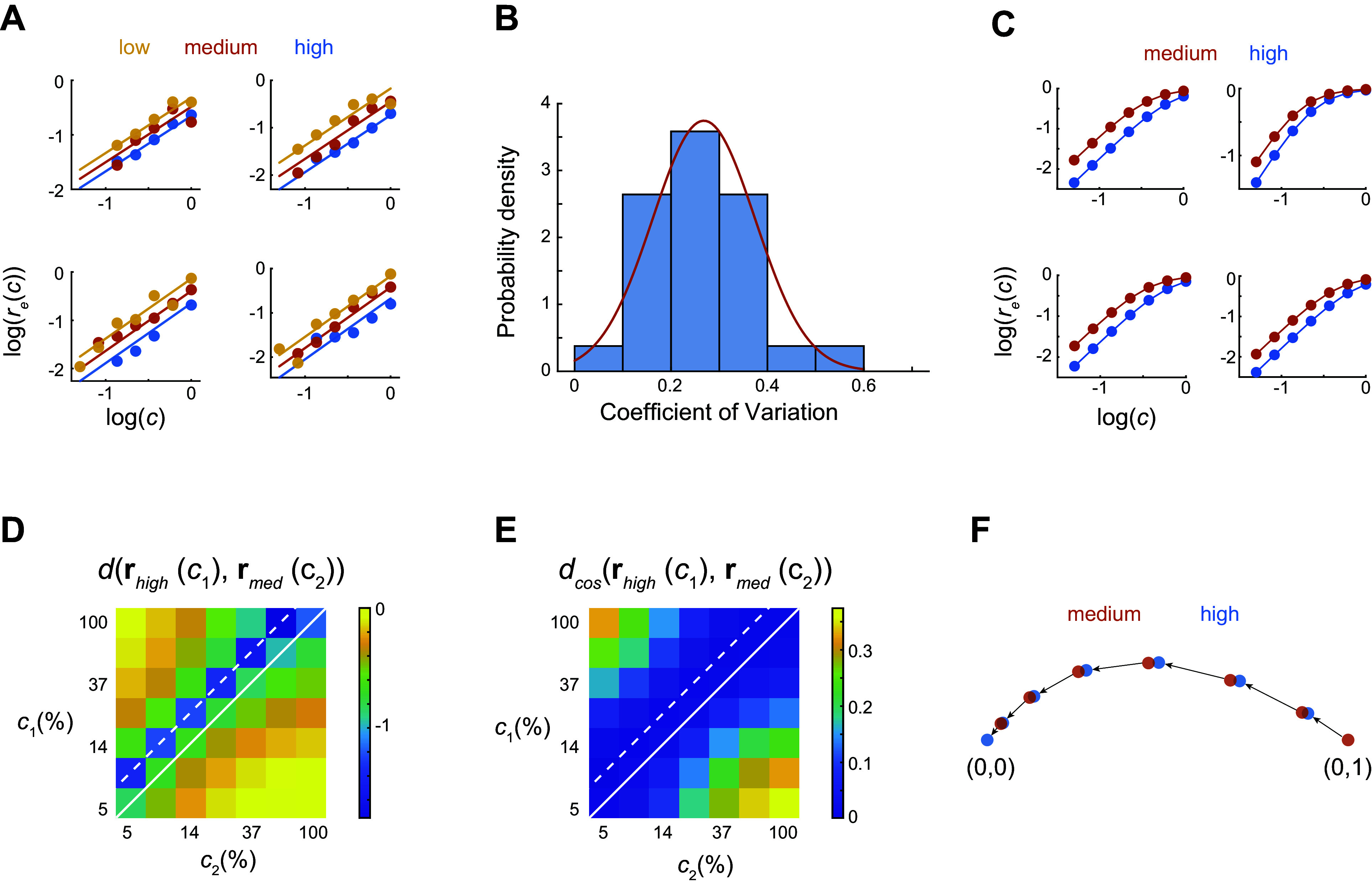
Observed dispersion in gain control across V1 neurons is consistent with reparameterization. *A*: example of contrast-response functions of single neurons for a fixed orientation. The solid lines are the fits of the model. The horizontal shift between the responses in different environments yields an estimate of the change in gain control. To assess the dispersion of gain control, we compute the coefficient of variation (CV) of the shifts in a population of neurons. *B*: distribution of the coefficient of variation pooled across different “arms” and imaging sessions. Only arms that included at least 3 responsive neurons in an arm were used. The coefficient of variation was corrected for bias given the small numbers involved, such that $\text{CV}=\left(1+\frac{1}{4n}\right){\text{CV}}_{\text{raw}}$, where *n* is the number of data points (20). The red curve represents a Gaussian fit. *C*: simulated neurons with Naka–Rushton response profile and a gain shift distributed to match the mean coefficient of variation in *B*. *D* and *E*: the structure of the Euclidean (*D*) and cosine (*E*) distance matrices recapitulates the findings observed in the population analysis, with the minimum distances lying along a diagonal displaced from the unity line. *F*: the first 2 principal components capture 99% of the variance in the data, have a similar shape to those observed experimentally, and illustrate that a change in the mean contrast of the environment causes responses to shift along a single curve, consistent with reparameterization (Fig. 1).

For any given session, we analyzed data obtained at a single orientation: one of the arms. In each arm, only a small fraction of cells will have a preferred orientation matching that of the stimulus and respond vigorously. Many other neurons respond with weak and noisy responses. If we analyze the contrast-response functions of the responsive neurons, we see that some recapitulate, at least partially, what is observed at the population level (Fig. 7*A*) (20). One common departure is that responses saturate at the highest contrast levels for the low-contrast environment (Fig. 7*A, top*, yellow data points). Nonetheless, the responses are reasonably fit by the model ${\hat{r}}_{e}\left(c\right)=A\;{\left(\frac{c}{{\bar{c}}_{e}^{\epsilon }}\right)}^{\delta }$, which allows us to estimate the shift in the contrast-response function of each neuron across environments (which equals ϵ log 2, as the mean contrast between the environments differ by 1 octave). We can then quantify the dispersion in the shifts by the coefficient of variation (CV) of ϵ. When pooling data across different arms and different experimental sessions, we find that, on average, CV = 0.27 ± 0.10 (mean ± 1 SD, computed over 53 different arms) (Fig. 7*B*).

Is such a degree of variability small enough for the population to approximate reparameterization? We know the answer must be affirmative, as this is what the population analysis shows. Nonetheless, we can further corroborate this expectation with a simple exercise. We simulate a population of neurons in one environment by assuming that the contrast response of the *i*th neuron is given by the Naka–Rushton equation $r_{i}\left(c\right)=c^{2}/(\sigma_{i}^{2}+c^{2})$ (6, 21). We selected σ*_i_* to be distributed as a Beta function with *a* = 3 and *b* = 6, to match approximately the distribution seen in V1 (22). We model a switch to a new environment of higher mean contrast by reducing the semisaturation constants by a factor drawn from a triangular distribution designed to match the coefficient of variation of the data (Fig. 7*C*). Now that we have contrast-response functions in the two environments, we can generate synthetic data and perform the same analyses as before, studying the structure of the pairwise Euclidean distance matrix (Fig. 7*D*) and the structure of the pairwise cosine distance matrix (Fig. 7*E*) and generating a low-dimensional visualization of the transformation of responses resulting from a switch of environments (Fig. 7*F*). We find that, for the experimentally observed degree of dispersion in gain control, the model reproduces the main phenomena seen in our data, including the shift of responses along a single curve, which we accepted as the signature of reparameterization (Fig. 7*F*). Thus, a moderate level of agreement between the changes in gain of neurons is sufficient to generate a representation that approximates reparameterization of responses by a population.

## DISCUSSION

The central aim of this study was to test the hypothesis that contrast gain control can be interpreted as a linear reparameterization of the contrast-response function of the population (Fig. 1*B*). Altogether, our data provide good support for this notion. First, a low-dimensional visualization of the population responses indicated that responses at a fixed orientation as a function of contrast lie along a single arm when switching between environments (Fig. 2). Second, analyzing the data separately for each arm showed that the response curves can be embedded in a plane and that the responses to different contrast levels shift along a single curve as the population as we modify the mean contrast of the environment (Fig. 3). As these findings rely on analyses performed after dimensionality reduction, we sought to reveal signatures of reparameterization in the native response space as well. In this context, we demonstrated that the responses in one environment can be matched by scaling the contrast in a second one, as shown by minima of pairwise distance matrices lying along a diagonal displaced from the identity line (Fig. 4). Finally, the data were well fit by a population contrast-response function that is a power law of contrast (13), where contrast gain is a power law of the geometric mean of the environment (Fig. 5). Such power law behavior emerges naturally in a population with a wide distribution of semisaturation constants. Altogether, our findings show that population responses under gain control can be viewed as the linear reparameterization **r**(*g_e_c*), with $g_{e}=\frac{1}{{\bar{c}}_{e}^{\epsilon }}$.

Reparameterization simplifies the job of downstream areas, as the identity of a visual pattern is represented by its contrast-response curve, which is invariant to changes in the distribution of contrasts in the environment. Finally, we note that perfect adaptation occurs for ϵ = 1. Our measured estimate of ϵ = 0.68 ± 0.06 means that, under the condition of our experiments, adaptation is only attained partially. Thus, coding of contrast is neither absolute (ϵ = 0) nor purely relative to the mean (ϵ = 1). We conjecture that values of ϵ closer to 1 may be possible if we restrict contrast distributions in the experiments to those observed naturally (23), rather than the synthetic distributions we used. The synthetic distributions used are highly unnatural, such as the high-contrast environment, where the most prevalent distributions are of high contrast rather than low (23).

In retrospect, a limitation of the study was the use of 7 log steps from 5% to 100% to sample the contrast domain. This choice left the range of contrast from 60% to 100% undersampled, which turned out to be the location where the contrast-response function appears to have the highest curvature (Fig. 2). Taking a more detailed look at the shape of the contrast-response curve calls for a denser sampling of contrast values, which will be remedied in future studies. Our findings are also limited to the truncated, log-normal distributions of contrast employed in these experiments (Fig. 1*C*). It will be important to extend the range of environments by using distributions found in natural scenes to verify that the behavior can be generalized (23, 24), including conditions where both contrast and mean luminance change (23, 25). In addition, in the present experiments we used a large, circular window for stimulation, which covered all the receptive fields of the population. Investigating the dependence of contrast gain with the spatial distribution of contrasts in the image is another important step in future studies (12, 19, 26, 27). Such data may help link reparameterization to illusions of perceived contrast, such as the effect observed in the simultaneous contrast illusion (11, 28). Finally, some of the details of the geometry we recovered may be distorted by nonlinear relationship between the actual spiking of neurons and our indirect inference from calcium imaging (29). High-density electrophysiology will need to be conducted to assess any possible departures from imaging data.

We have not yet delved into the neural mechanisms implementing reparameterization, but this would have to address how the network can generate well-matched changes of gain in a local population (38, 39, 40, 41). One prominent candidate is using a pooled cortical signal that controls gain in the local population (5) rather than by independent, self-calibration (30). The ability to influence a pooled signal could also serve as a central “knob” that accounts for a separable power law relationship between the magnitude of the response and the probability of stimulus (31), its contrast (13), and, as shown in the present study, the mean contrast of the environment. Each of these factors appears to tweak the same gain knob, as demonstrated by the fact that a change in one can be compensated for by a change in another. For example, an increase in the probability of a stimulus can be counteracted by an increase in its contrast to keep the response magnitude constant (13).

The findings of a prior study reported that the direction of the population vector to a stimulus of a fixed orientation is invariant with contrast, such that **r**(θ,*c*) = **h**(θ)*f*(*c*) (32). In our data, an approximate relationship is observed at low to moderate contrasts, where the arms appear close to rays emanating from the origin, as implied by the above factorization of the population response into a vector response to orientation and a scalar function of contrast. However, the full extent of the contrast-response curve shows substantial curvature (Fig. 2 and Fig. 3) (see also Ref. 33). Technical differences between the studies may account for this apparent discrepancy. Namely, Busse et al. (32) recorded multiunit activity in an electrode array and defined population responses by first grouping signals across electrodes according to their preferred orientation and averaging their responses. The population vector was defined based on such “average neurons.” As the authors show, if the distribution of the semisaturation constant is independent of the shape of orientation tuning curves (as they observed), such averaging would necessarily lead to the factorization of orientation and contrast at the population level. In contrast, we define a population response as a vector where each entry represents the activity of a single neuron. In this scenario, separability between orientation and contrast occurs if the responses of each neuron are separable themselves and if they all share the same contrast-response function. The first condition holds and is well known as the contrast invariance of orientation tuning in single neurons (34–36); the second condition does not hold, as neurons with a preference for a particular orientation have a diversity of contrast-response functions. This alone means that contrast invariance at the population level is not possible (33) but may be obtained by averaging the responses of neurons with similar tuning preferences (32).

We close by noting that the geometric view of contrast gain control is nothing more than a convenient way to look at the average behavior of a large population of neurons as they adapt to the contrast of an environment. The behavior of the population simply reflects the collective behavior of individual neurons. Other than a mechanism that leads to well-matched changes in the gain of neurons, there are no other “emergent” phenomena demonstrated by our data. The advantage of our analyses is that it reveals lawful statistical relationships that describe the population responses accurately, even though many individual neurons respond weakly and generate noisy responses (37). An apt analogy would be a description of the behavior of gases in terms of statistical quantities such as pressure, temperature, and volume. Here, we can obtain simple relationships between these quantities even though the behavior of the individual molecules can show considerable variability.

## DATA AVAILABILITY

Raw data for these experiments, along with code to access them, have been deposited in a Figshare repository: https://doi.org/10.6084/m9.figshare.26999737.v1.

## GRANTS

This work was supported by Grants EY035064 (D.L.R. and M.D.), NS116471 (D.L.R.), and EY034488 (D.L.R.).

## DISCLOSURES

D.L.R. has a financial interest in Scanbox imaging electronics and software. None of the other authors has any conflicts of interest, financial or otherwise, to disclose.

## AUTHOR CONTRIBUTIONS

D.L.R. conceived and designed research; E.T. and D.L.R. performed experiments; D.L.R. analyzed data; S.A.M., M.D., and D.L.R. interpreted results of experiments; D.L.R. prepared figures; D.L.R. drafted manuscript; S.A.M., M.D., and D.L.R. edited and revised manuscript; M.D. and D.L.R. approved final version of manuscript
